# *In situ* growing 3D-Cu coating to improve the reversibility and reaction kinetics of Zn metal anodes

**DOI:** 10.3389/fchem.2022.1037995

**Published:** 2022-10-12

**Authors:** Lianbao Liang, Lifeng Hang, Shuangcong Xie, Dandan Men, Guihua Jiang, Yiyu Chen

**Affiliations:** ^1^ Department of Medical Imaging, Guangdong Second Provincial General Hospital, Guangzhou, China; ^2^ Shanxi Province Key Laboratory of Microstructure Functional Materials Institute of Solid State Physics, Shanxi Datong University, Datong, China

**Keywords:** zinc metal anode, 3D-Cu alloy coating, aqueous Zn-based battery, alloying–dealloying process, MnO_2_–Zn

## Abstract

The zinc metal anode is the most promising metal anode material in aqueous battery systems due to its low cost and high theoretical capacity. However, it still undergoes irreversible reactions such as premature failure of the dendrites/dead Zn during Zn stripping/plating, resulting in the inferior cycling stability of the Zn-based full cell. Here, we demonstrate a facile 3D-Cu alloy coating to improve Zn reversibility by providing spatial voids to accommodate the plated Zn to form dendrite-free morphology. Combining the larger 3D surface and the alloying–dealloying process, the Zn anode reactions exhibit enhanced reaction kinetics to meet large operating current densities. The 3D-Cu-coated Zn anode can deliver improved cycling stability for 350 h under a large areal capacity of 3 mAh cm^−2^. It also enables MnO_2_–Zn at the full cell level to achieve a specific capacity of 205 mAh g^−1^ and longer cycling for 350 cycles with 87.4% retention of the initial capacity. This research provides a new pathway to achieve high reversible Zn metal chemistry.

## 1 Introduction

High safety and low cost are the basic requirements for energy storage applications (Tang et al., 2019; Mo et al., 2020; Lv et al., 2022). However, commercial lithium-ion batteries (LIB) still face safety risks by utilizing organic electrolytes and the high cost of Li elements. Aqueous battery systems are a promising alternative for energy storage fields (Posada et al., 2017; Liang et al., 2021; Yang et al., 2022a), where Zn-ion batteries are widely investigated candidates by using Zn metal as the anode due to its high theoretical capacity (5855 Ah L^−1^ and 820 Ah kg^−1^) and low redox potential (0.76 V vs. the standard hydrogen electrode) (Zeng et al., 2019; Liu et al., 2020a; Chao et al., 2020; Yang et al., 2021; Yang et al., 2022b).

As for the Zn anode, improving its reversibility is very important to prolong the cycling stability of the zinc battery (Ma et al., 2020; Hao et al., 2021; Liang and Zhi, 2021). At present, there are three general strategies to promote Zn anode reversibility for zinc anode protection. The first is electrolyte engineering, generally by adding organic solvents/additives (Guo et al., 2021a; Cao et al., 2021; Hao et al., 2021), which is effective and feasible for scalable production. However, the introduction of organic solvents/additives may violate the intrinsic safety performance of aqueous electrolytes. The second is applying a 3D current collector by increasing the surface areas for Zn deposition to avoid the accumulation of Zn growth to form Zn dendrites (Yang et al., 2020; Guo et al., 2021b; Fan et al., 2021; Ni et al., 2022). However, it might lose the volumetric energy density of Zn-based batteries at the full-cell level. The third is adopting surface-protective coatings, where the coatings can be insulative inorganics and conductive metals/alloys. As for the insulative coatings, such as calcium carbonate (Kang et al., 2018) and titanium oxide (Li et al., 2021), even though they can hinder the direct generation of hydrogen evolution reactions (HERs), they will increase the interface resistance for poor power density with large voltage polarizations, particularly at high current densities. On the other hand, conductive metal/alloy coatings are verified to be conducive to reversible Zn not only by providing a larger specific surface area but also by increasing corrosion barriers. For example, the indium (In)-based InZn (Xiao et al., 2022), bismuth (Bi)-based BiZn (Wang et al., Forthcoming 2022), and gradient CuZn (Liang et al., 2022) alloy coatings for the Zn metal anode (ZMA) have been demonstrated to inhibit Zn dendrite formation and the HER at the same time during long-term Zn stripping/plating cycling. Thus, based on the excellent performance of conductive alloy coatings (Liu et al., 2021), it is highly desirable to explore novel Zn-based alloy anodes with highly compatible and stable electrode/electrolyte interfaces for high-performance AR-ZMBs.

In this report, we apply a feasible strategy *via* a chemical substitution reaction *in situ* to build a protective 3D-CuZn alloy layer. It is found that the reversibility of ZMA is largely improved by not only inhibiting Zn dendrites but also the HER issues. In particular, such improvement is attributed to the formation of a 3D framework of the protective ZnCu alloy layer to accommodate the deposited Zn, leading to dendrite-free morphology. In addition, the dendrite-free Zn morphology would in turn suppress hydrogen evolution, further contributing to the cycling stability of ZMA. Compared to the bare ZMA, the 3D-Cu-coated Zn anode has delivered 350-h cycling stability with a high areal capacity of 3 mAh cm^−2^ at a current density of 3 mA cm^−2^. When paired with MnO_2_ cathodes, the full cells can deliver a capacity of 242 mA h g^−1^ at 1 mA cm^−2^, and the capacity retention is 87.4% after 350 cycles at a high current density of 5 mA cm^−2^. These results indicate the effectiveness of the 3D-CuZn alloy coating for improving highly stable Zn metal batteries.

## 2 Results

**FIGURE 1:**
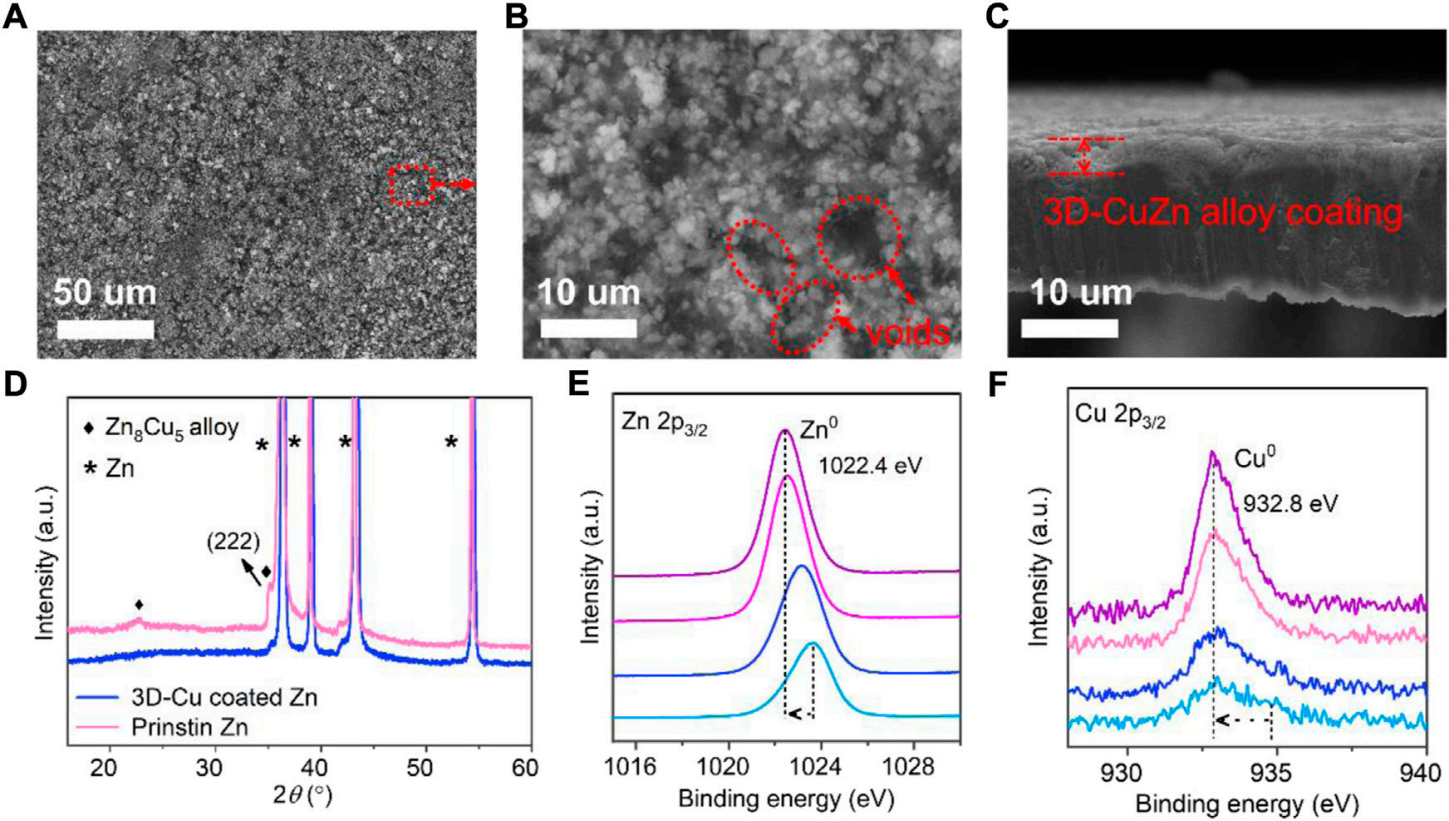
Characterizations of 3D-Cu-coated electrodes and bare Zn. **(A)** SEM image and **(B)** magnified image from the marked-out region in **(A)**. **(C)** Cross-sectional SEM image of the 3D-Cu-coated electrodes. **(D)** XRD result of these two Zn electrodes. **(E,F)** XPS results on the depth etching profiles of Zn and Cu, where the arrow points from the initial state to the final state throughout the etching process, respectively.

The 3D-Cu-coated Zn was *in situ* prepared by a facile procedure by immersing bare Zn in 0.1 M CuSO_4_ solution, while the Zn metal would get substituted by Cu^2+^ ions to produce the ZnCu alloy phase (Chen et al., 2021; Xiao et al., 2022). It was verified by the X-ray diffraction (XRD) results with the peak at 36.5° as the (222) peak of the Zn_8_Cu_5_ alloy phase (JCPDS: 025-1228), verifying the formation of the ZnCu alloy (Figure 1A). On the other hand, the geometric depth profiles of the X-ray photoelectron spectroscopy (XPS) results revealed the shift of the Zn 2p3/2 peak from a higher binding energy to lower values (Figure 1B), while the intensity of Cu is increased but the binding energy is barely shifted (Figure 1C). It might be a result from the variations of the valence states of surficial Zn when alloying with the Cu metal (Liang et al., 2022).

Furthermore, a scanning electron microscope (SEM) was used to observe the morphology of bare Zn and ZnCu alloy-coated Zn. On bare Zn, irregular defects/cracks existed (Supplementary Figure S1), which can result in an uneven electric field distribution during Zn deposition, promoting Zn dendrite formation. There are spatial voids existing among the nanoparticle shapes of the ZnCu alloy as the 3D frameworks of CuZn on Zn, named 3D-Cu-coated Zn, which is conducive to accommodating the deposited Zn to form dendrite-free morphology (Liu et al., 2020b; Xie et al., 2020; Lu et al., 2021) (Figures 1D,E). In addition, the enlarged surface area of 3D frameworks can provide more contact areas for fast dynamics of the Zn anode to meet large current densities. The contact angle of bare Zn and 3D-Cu-coated Zn electrodes with 1 M ZnSO_4_ electrolyte was exhibited (Supplementary Figure S2). In particular, the 3D-Cu-coated Zn featured superior wettability with a contact angle of 33°, while the bare Zn electrode had a contact angle of 84°, indicating better electrolyte affinity for fast Zn-ion nucleation. The thickness of the 3D-CuZn coating is about 3 μm according to the cross-sectional SEM (Figure 1F), and it can provide sufficient space for the deposited Zn.

**FIGURE 2:**
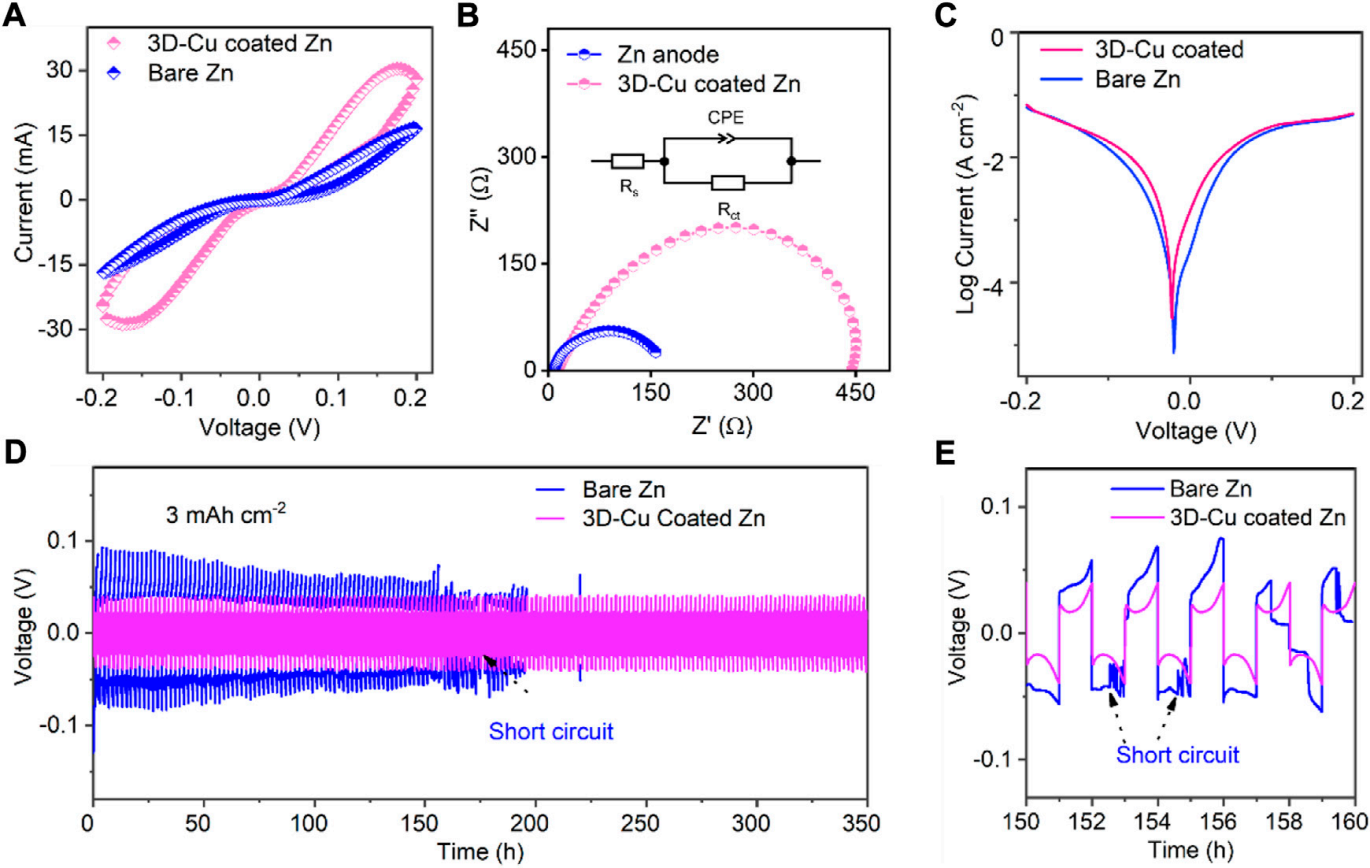
Electrochemical performance of 3D-Cu-coated electrodes and bare Zn in symmetric cells. **(A)** CV results at a scanning rate of 5 mV s^−1^. **(B)** EIS results of the symmetric cells at initial states and the inset is the equivalent circuit, where R_s_ represents electric resistance, and R_ct_ is the charge transfer resistance. **(C)** Tafel results of symmetric cells. **(D)** Plating/stripping results at 3 mA cm^−2^ with a capacity of 3 mAh cm^−2^. **(E)** Selected voltage profiles to observe the short circuit of the cell based on bare Zn.

A set of electrochemical tests was conducted to study the performance of the 3D-Cu-coated Zn by comparing it with the performance of bare Zn without modification. First, there is a significant difference between the cyclic voltammetry (CV) curves of bare Zn and 3D-Cu-coated Zn in symmetric cell configuration (Figure 2A), where there is a pair of redox peaks for the 3D-Cu-coated Zn but absent for bare Zn. These CV peaks can be attributed to the alloying–dealloying process between the Zn metal and Cu metal, which is different from the general plating/stripping process on bare Zn. Moreover, the specific current density of 3D-Cu-coated Zn is about two-fold larger than that of bare Zn, indicating better kinetics for Zn plating/stripping on 3D-Cu-coated Zn. In addition, electrochemical impedance spectroscopy (EIS) of the symmetric cells also verified the facile and faster mass transfer with lower charge transfer resistance of 153.5 Ω for 3D-Cu-coated Zn, which is much smaller than that of bare Zn as 447.8 Ω (Figure 2B). The initial resistances of these two cells are comparable, as shown in Supplementary Table S1. Last, the Tafel profile was tested, and it showed that the corrosive potential is F02D19 mV for 3D-Cu-coated Zn, which is at a more positive potential than the bare Zn electrode as −24 mV, exhibiting a higher barrier of CuZn for corrosion and accommodating stable reaction processes against corrosion with hydrogen evolution. The cathodic Tafel slope is larger with 105.4 mV dec^−1^ for the 3D-Cu-coated Zn than 73.7 mV dec^−1^, featuring faster kinetics of the 3D-Cu-coated Zn.

To highlight the advantages of 3D-Cu-coated Zn, we used a large surface capacity of 3 mAh cm^−2^ to test the stability of the 3D-Cu-coated Zn electrode, which can show a stability of 350 h. On the other hand, for the bare Zn electrode, a short circuit occurred in 153 h (Figure 2D). It shows that the coating is beneficial to the cycle stability under large surface capacity. The galvanostatic charging–discharging (GCD) curves of the 3D-Cu-coated Zn and bare Zn electrodes are shown in Figure 2E, and the curve of the 3D-Cu-coated Zn electrode is stable and consistent while it begins to fluctuate at the 77th cycle for premature cell failure. In addition, the polarization voltage of coating is 47 mV, while that of bare Zn is 85 mV, and the smaller polarization voltage indicates the faster dynamics of the Zn plating/stripping process (Figure 2E).

**FIGURE 3:**
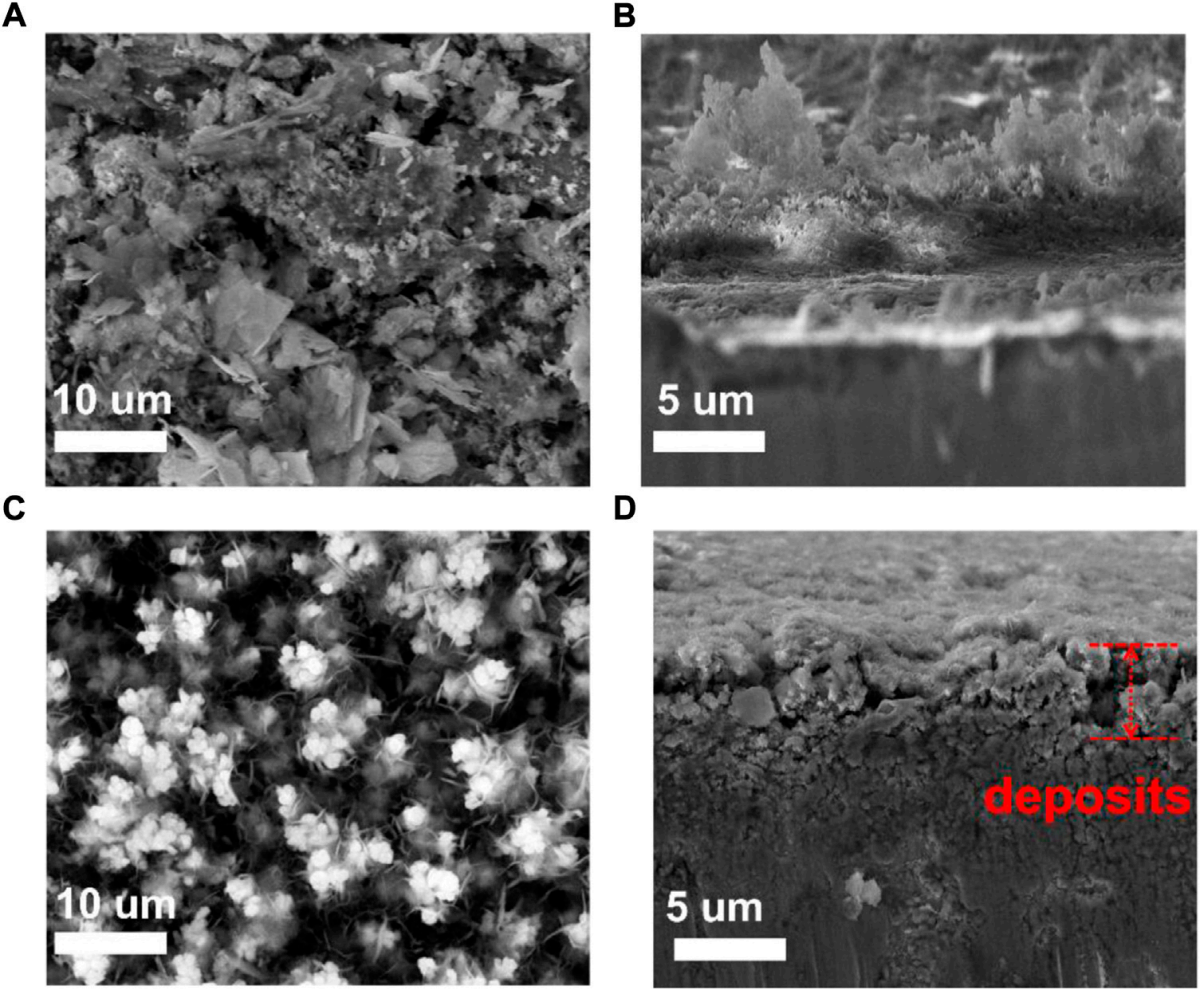
SEM images and the cross-sectional images of bare Zn and 3D-Cu-coated electrodes after deposition at 3 mAh cm^−2^. **(A** **,B)** Bare Zn electrode. **(C,D)** 3D-Cu-coated electrodes.

The morphology of the Zn anode at deposited states after cycling for 20 cycles was characterized, and the surface and cross-section images are shown in Figure 3. Dendritic Zn flake morphology is formed for bare Zn, as shown on the top and cross-sectional morphology, which can lead to the possibility of a short circuit. On the other hand, the voids between Cu particles of the 3D-Cu coating were filled by the deposited Zn, and there is no Zn dendrite growth (Figures 3C,D). This shows that 3D Cu coating inhibits dendrite growth.

**FIGURE 4:**
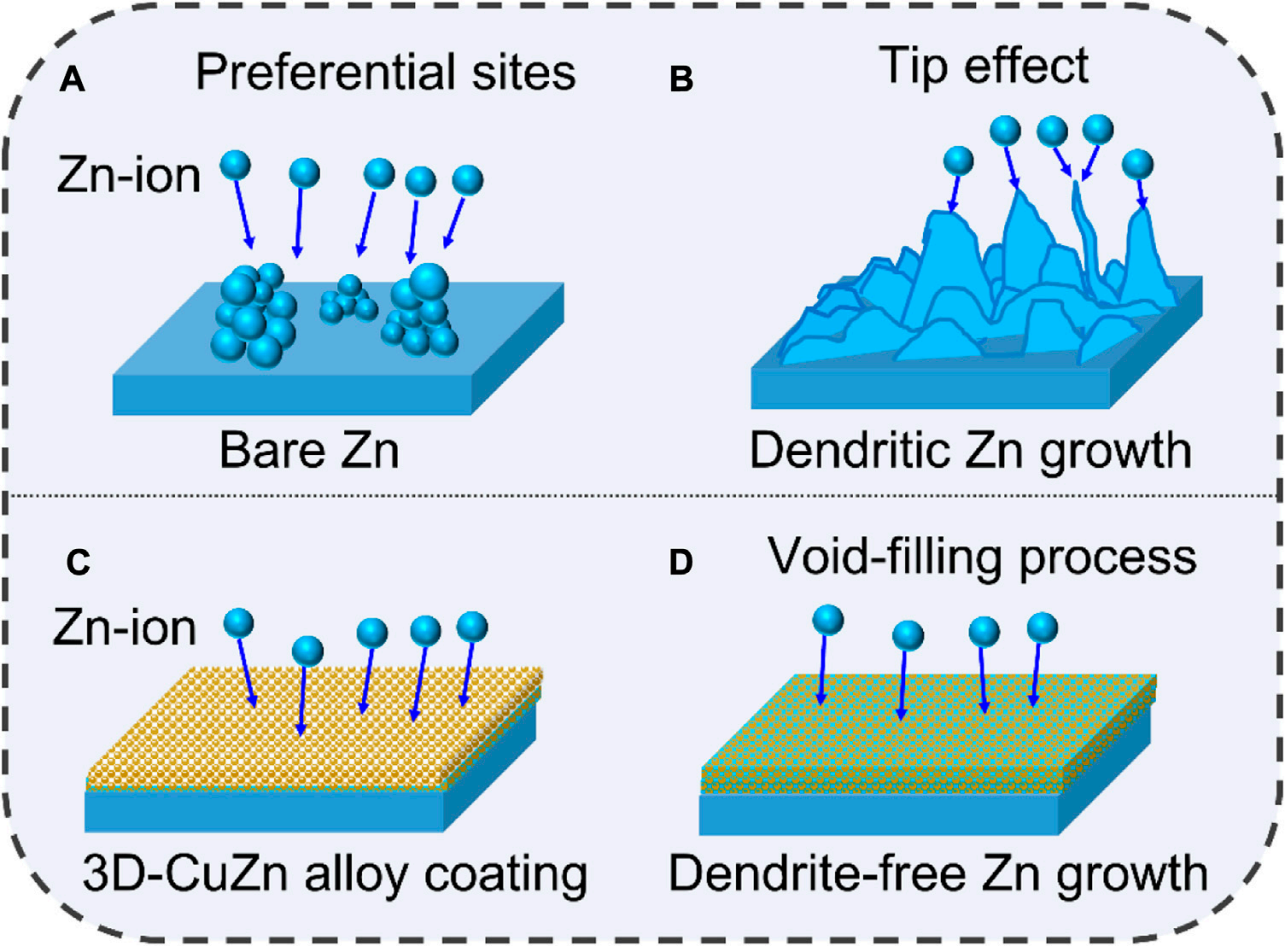
Zn growth schematics of bare Zn and 3D-Cu-coated electrodes. **(A,B)** Bare Zn electrode. **(C,D)** 3D-Cu-coated electrodes.

The corresponding Zn plating processes on bare Zn and 3D-Cu-coated Zn are proposed as shown in Figure 4. The Zn ions would first nucleate at preferential spots on the bare Zn, such as defects/cracks, and then accumulate to grow at these spots to form dendrites. It would further promote the growth of Zn dendrites due to a tipping effect by the concentrated electric field (Figures 4A,B). On the other hand, the Cu nanoparticles can serve as deposition sites to accommodate the deposited Zn in the alloying process. In addition, the voids can accommodate Zn to promote Zn dendrite-free morphology without Zn protrusion (Figures 4C,D).

**FIGURE 5:**
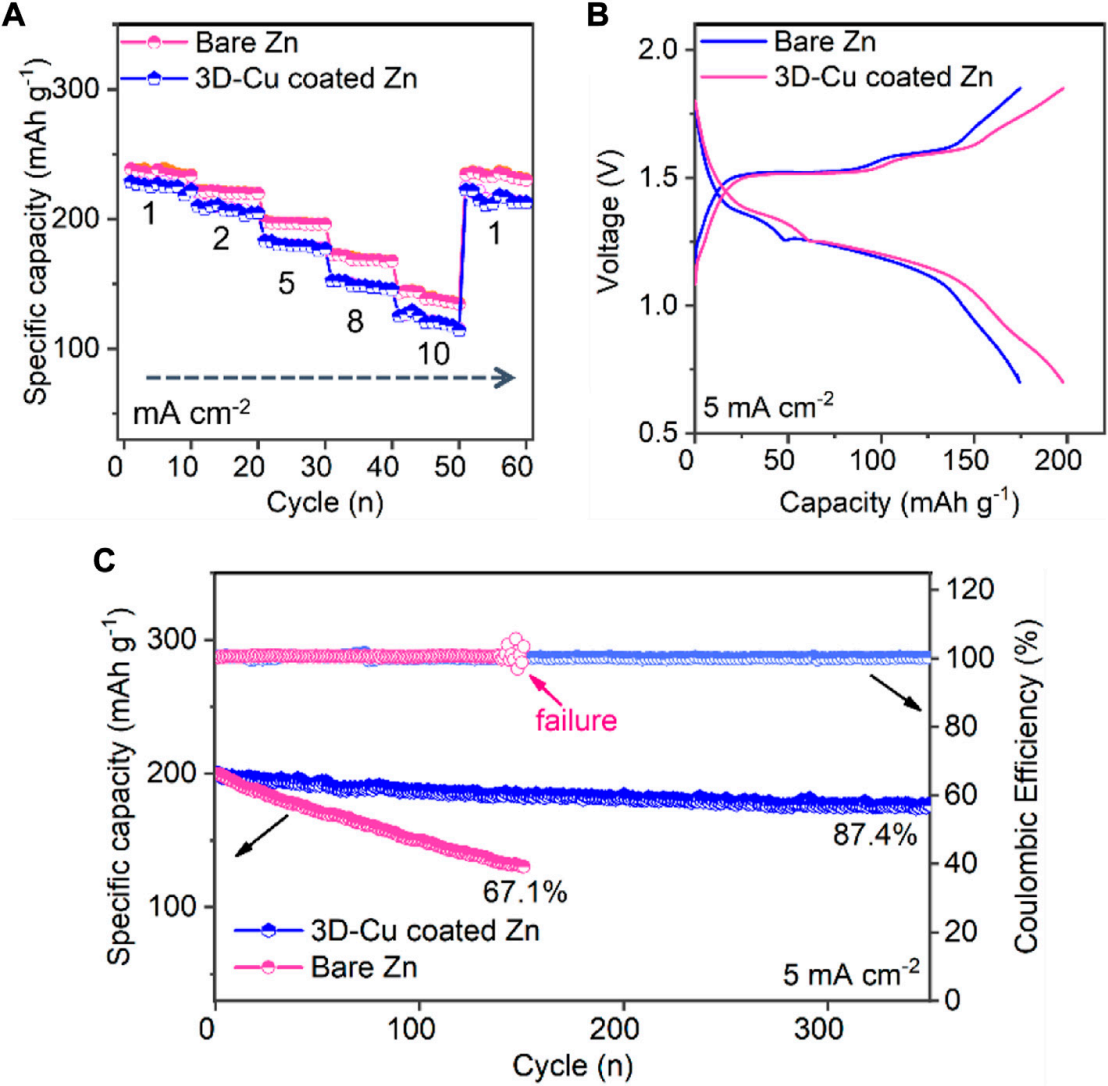
Electrochemical performance of MnO_2_–Zn full cells based on bare Zn and 3D-Cu-coated electrodes. **(A)** Rate performance at the current densities from 1 to 10 mA cm^−2^. **(B)** GCD profiles at 5 mA cm^−2^. **(C)** Cycling performance.

The full Zn–MnO_2_ cells were fabricated to test the performance of these two Zn anodes. The 3D-Cu-coated Zn anode exhibited a better rate of performance than bare Zn. In particular, the specific capacity is 247 mAh g^−1^ at a lower current density of 1 mA cm^−2^ for 3D-Cu-coated Zn, and the capacity is 151 mAh g^−1^ at a high current density of 10 mA cm^−2^. On the other hand, the capacity of bare Zn is comparable to that of the 3D-Cu-coated Zn anode at 241 mAh g^−1^ at a low current density of 1 mA cm^−2^, but it is much smaller at 113 mAh g^−1^ at a high current density of 10 mA cm^−2^. It indicates that the 3D-Cu coating endows fast kinetics for the Zn anode at full-cell levels due to the larger specific surface area and the fast alloying/dealloying process. The corresponding GCD curves of these two full cells are shown in Figure 5B, where the cell based on the 3D-Cu-coated Zn was featured with smaller polarization voltage and larger specific capacity. The origin of the smaller polarization of GCD profiles of the full cell can be ascribed to the smaller potential polarization of the Zn anode, where the 3D Cu anode can provide faster kinetics with smaller polarization, as evidenced by Figure 2B. Finally, during the long cycle of the whole battery, the capacity of bare Zn dropped to 67.1% after 144 cycles, and there was a premature failure with a fluctuation in Coulombic efficiency. This 3D-Cu-coated Zn anode can demonstrate an initial capacity at 205 mAh g^−1^ and longer cycling of 350 cycles with 87.4% retention of the initial capacity (Figure 5C).

## 3 Discussion

In order to improve the reversibility of ZMA, 3D-Cu coating was applied onto the Zn surface to act as an effective protective layer. Unlike other conductive coatings, the Zn plating–stripping process corresponds to the alloying–dealloying process, and there are sufficient structural voids to provide enhanced contact areas between the anode and the electrolyte, jointly improving the reaction kinetics of ZMA. In addition, these voids can provide space for the deposited Zn, which can largely inhibit the growth of Zn dendrites to obtain dendrite-free morphology. The formation of the Cu–Zn alloy also increases its corrosion potential. The dendrite-free morphology and anti-corrosive property jointly inhibit HERs together and improve ZMA’s reversibility.

Our findings provide new insights into the metal coating strategy for ZMA, especially with the capability to proceed the alloying–dealloying process with ZMA. Therefore, metals, such as tin, In, and Bi, can be further applied as potential candidates for metal coatings to improve Zn reversibility. This report can also provide guidance for the coating structure design of the ZMA surface, and the efficient 3D structure of the designed 3D metal coating is of positive significance for coating strategies.

## 4 Conclusion

A simple method has been developed to stabilize ZMA by coating a layer of the CuZn alloy on the surface of the Zn anode. The as-obtained 3D-Cu coating structure can provide many voids which can endow dendrite-free morphology to promote more stable reversibility and cyclic stability of ZMA. Moreover, such a coating can accommodate the alloying–dealloying process to endow faster kinetics. The reversibility and fast kinetics of the zinc anode can correspond to the stability of the whole battery. The metal coating strategy provides a model for other metal–alloy coatings to further achieve more stable Zn anode reversibility.

## Data Availability

The original contributions presented in the study are included in the article/Supplementary Material; further inquiries can be directed to the corresponding authors.
