# Supplementary material for: In situ growing 3D-Cu coating to improve the reversibility and reaction kinetics of Zn metal anodes
Source: Front Chem. 2022 Oct 12;10:1037995. doi: 10.3389/fchem.2022.1037995 (PMC9597878; doi:10.3389/fchem.2022.1037995)
Supplement: Supplementary file 1 [file DataSheet1.docx]

**Supplementary Materials**

**In-situ Growing 3D-Cu Coating to Improve the Reversibility and Reaction Kinetics of Zn Metal Anode**

Lianbao Liang^1^, Lifeng Hang^1,*^ Shuangcong Xie^1^, Dandan Men^2^, Guihua Jiang^1^, Yiyu Chen^1,*^

1 Department of Medical Imaging, Guangdong Second Provincial General Hospital, Guangzhou 518037, China

2 Shanxi Province Key Laboratory of Microstructure Functional Materials Institute of Solid State Physics, Shanxi Datong University, Datong, 037009, China

*Correspondence: hanglf@ustc.edu.cn; chenyy@gd2h.org.cn

**Experimental Section
*Preparation of 3D-Cu coated Zn Electrode***The Zn foil of 50 μm thickness was first washed in by water and ethanol, and then the Zn foil were immersed in CuSO_4_ solution (50 mM) for 30 s to obtain 3D-Cu coated Zn electrodes through a chemical substitution reaction. The as‑prepared 3D-Cu coating were further washed in pure water to remove residual ions on the surface and then directly utilized in symmetric and full cells.

***Preparation of MnO_2_ electrode***

The MnO_2_ cathode were prepared by a hydrothermal method. In specific, the mixture of KMnO_4_ (80 mM)and MnCl_2_ (45 mM) was added into a Tefon‑lined steel autoclave and then was heated at 180 °C for 12 h in an oven. Then precipitates were further washed in pure water for several times and dried in a vacuum oven at 40 °C. The MnO_2_ cathode was prepared by mixing the as‑obtained MnO2 with acetylene black and poly(vinylidene difuoride) with a with weight ratios of 8:1:1 in N‑methyl‑2‑pyrrolidone solvent, and then pasted it on titanium foil with the loading mass of 1.5 mg cm^-2^.

***Electrochemical Characterizations***

Symmetric cells and full cells were assembled in CR-2032 coin cells by applying bare Zn and 3D-Cu coated Zn, respectively, while the a volume of 150 µL of electrolyte (1 M ZnSO4) was utilized with the glass fiber (Waterman-1820) as separator. Cyclic voltammetry and Tafel plot were measured using a CHI-760 electrochemical working station. Electrochemical impedance spectroscopy (EIS) measurements of symmetric cells were taken over the frequency ranging from 100 kHz to 0.1 Hz with an amplitude of 5 mV. Galvanostatic cycling of anode stripping/plating behaviors of 3D-Cu coated Zn and Zn were tested at current density of 3 mA cm^-2^ and the capacity of 3 mAh cm^-2^ using the CT2001A Battery Cycler (Wuhan,China). The rate capability and cycling performance of full cells based on the bare Zn and 3D-Cu coated Zn was tested on CT2001A Battery Cycler (Wuhan,China).

***Materials Characterizations***

X‑ray diffraction (XRD) measurements were performed on Bruker D2 Phaser and the X‑ray photoelectron spectroscopy (XPS) analysis was conducted on Thermo ECSALAB 250 with etching for 180 seconds with 60 second duration. The SEM was performed on (SEM–EDS, JEOL,JSM‑7900F, 15 kV).


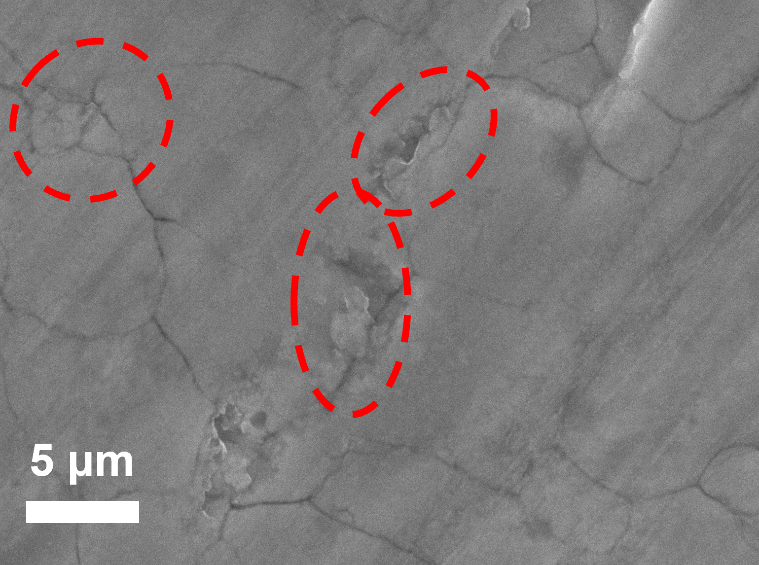


**Figure S1.** SEM image of the pristine bare Zn electrode with the cracks/defects marked out.


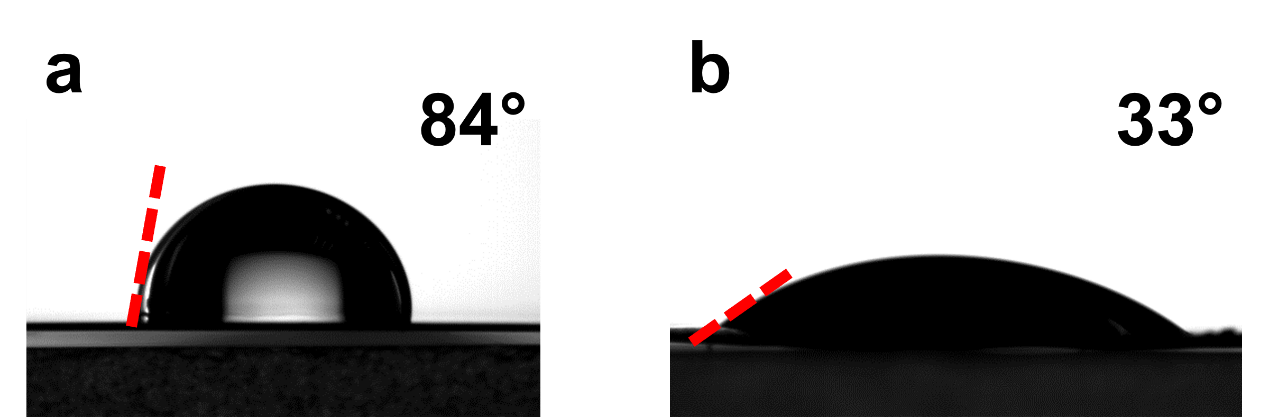


**Figure S2.** The contact angle tests of the bare Zn (a) and the 3D-Cu coated Zn.
